# On evaluating the efficiency of the delta-lognormal mean estimator and predictor

**DOI:** 10.1016/j.mex.2022.101830

**Published:** 2022-08-23

**Authors:** Philippe Aubry

**Affiliations:** OFB - Office français de la biodiversité - Direction surveillance, évaluation, données - Unité données et appui méthodologique, Saint Benoist, BP 20, Le Perray-en-Yvelines F-78612, France

**Keywords:** Delta-lognormal distribution, Superpopulation, Point estimation, Finite population, Mean model, Empirical predictor, Prediction error variance, Relative efficiency, Uniformly minimum-variance unbiased estimator (UMVUE), Confluent hypergeometric limit function

## Abstract

A variable taking positive values from a lognormal distribution and null values with a given probability is distributed according to the so-called delta-lognormal distribution. Two situations arise depending on whether the data are regarded as a random sample from an infinite population (superpopulation) or from a finite population, itself considered as a random sample from a superpopulation. In the case of an infinite population, estimating the mean can be accomplished using a uniformly minimum-variance unbiased estimator (UMVUE). Likewise, the prediction of the mean in the case of a finite population may be based on the UMVUE. In both cases, one expects a gain in precision when taking into account the shape of the distribution by relying on the UMVUE rather than on the sample mean, which is a nonparametric estimator (or predictor).

1. For the infinite population case, the relative efficiency results presented in this article are more complete and more accurate than those published so far.
2. The article fills a gap regarding the question of relative efficiency in the case of a finite population.
3. Calculations were performed using the exact expression for the variance of the UMVUE of the mean, expressed in terms of the confluent hypergeometric limit function.

## Graphical abstract

**Figure:**
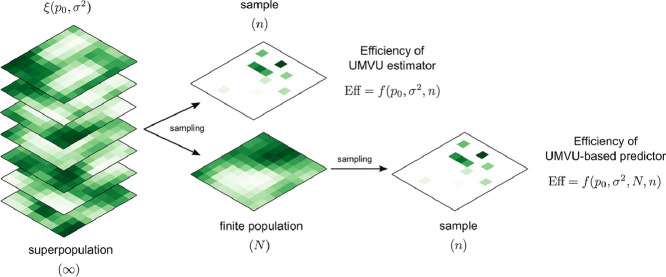

Specifications table

**Table:** 

Subject area:	Probability distributions
More specific subject area:	Relative efficiency of statistical estimators or predictors
Method name:	UMVU-based estimation or prediction of the mean for delta-lognormal data
Name and reference of original method:	• J. Aitchison, J. Brown, The lognormal distribution, Cambridge University Press, Cambridge, UK, 1957.
	• K. Shimizu, Point estimation, in: E. L. Crow, K. Shimizu (Eds.), Lognormal distributions: theory and applications, Marcel Dekker, New York, USA, 1988, pp. 27-86.
	• S. Smith, Evaluating the efficiency of the Δ-distribution mean estimator, Biometrics 44 (1988) 485-493.
Resource availability:	*not applicable*

## Method details

Let U be a finite population of sampling units, unambiguously identifiable by integer labels i=1,2,⋯,N. Let y be a variable of interest measured or observed on the sampling units, and the total $t_{U}=N{\bar{y}}_{U}$, with ${\bar{y}}_{U}$ the mean of y defined over U. A sample s⊆U of size n is drawn from U by an ignorable selection mechanism. Classical (frequentist) statistics assume that the $y_{i}$ (i∈U) are random variables of joint distribution ξ. Said another way, U is itself a random sample drawn from an infinite set of populations sharing the same general statistical properties (i.e., a superpopulation), described by stochastic model ξ. In this article, we refer to the situation where, from the sample s at hand, the purpose is either to estimate the expectation of y in the model ξ, or to predict the finite population mean ${\bar{y}}_{U}$ (or equivalently, the total $t_{U}$).

We consider here a variable of interest y taking nonnegative values (y≥0). The sample s can be partitioned into $s=s_{0}\cup s_{1}$, $s_{0}\cap s_{1}=\emptyset$, with $s_{0}$ of size $n_{0}$ having zero values (y=0) and $s_{1}$ of size $n_{1}$ having positive values (y>0). If $n_{1}=1$, we note the unique positive value $y_{s_{1}}$. A characteristic of such data is that they may exhibit a high proportion of zero values. To take this into account in a sufficiently flexible manner, one approach is to use a two-component mixture model. There are two possibilities: (i) increasing the probability of zero values from a distribution defined for y≥0 (*zero-inflated distributions*); (ii) introducing a dichotomy between y=0 and y>0 in a mixture model with two separately estimable parts (*hurdle-at-zero, conditional, two-part, and delta distributions* designate the same thing). If the second possibility is adopted, then a suitable model for nonnegative values is written as:

(1) $$G\left(y;p_{0},\theta \right)=\left\{ \begin{array}{cc} p_{0} & \;y=0\\ p_{0}+\left(1-p_{0}\right)F\left(y;\theta \right) & \;y>0 \end{array}\right.$$

where F(y;θ) is a cumulative distribution with parameters θ, corresponding to a positive distribution, either discrete or continuous; here we consider the lognormal distribution.

### Lognormal distribution

Let z be a random variable distributed according to the standard normal distribution (i.e., z∼Norm(0,1)) of probability density:

(2) $$\phi \left(z\right)=\frac{1}{\sqrt{2\pi }}\exp\left\{-\frac{1}{2}z^{2}\right\}\;\;z\in R$$

Then, y=exp(μ+σz) follows a lognormal distribution that is completely specified by μ and $\sigma^{2}$. The probability density function of the lognormal distribution is written as [1, p. 8, Eq. (2.5)]:

(3) $$f\left(y;\mu ,\sigma^{2}\right)=\frac{1}{y\sigma }\phi \left(\left(\ln y-\mu \right)/\sigma \right)=\frac{1}{y\sigma \sqrt{2\pi }}\exp\left\{-\frac{1}{2}{\left(\frac{\ln y-\mu }{\sigma }\right)}^{2}\right\}\;\;y\in R_{+}^{*}$$

### Delta-lognormal distribution

The lognormal distribution is no longer appropriate when zero values must be accounted for. This leads to using the delta-lognormal distribution [2], often also called the Δ*-distribution* [1] and occasionally the *Bernoulli-lognormal two-part model* (e.g., [3, p. 703]).

The delta-lognormal distribution results from a mixture of a Dirac mass at 0 with probability $p_{0}$ and a lognormal distribution with probability $\left(1-p_{0}\right)$, that is:

(4) $$g\left(y;p_{0},\mu ,\sigma^{2}\right)=p_{0}\;\delta \left(y\right)+\left(1-p_{0}\right)\;f\left(y;\mu ,\sigma^{2}\right)$$

where δ(y) is a Dirac distribution that concentrates a unit mass at 0.

The first three cumulants (i.e., expectation, variance, and third central moment) of the distribution (4) are written as [1, p. 95, Eq. (9.43)–(9.45)]:

(5) $$\kappa_{1}=(1-p_{0})\;\alpha$$

(6) $$\kappa_{2}=\left(1-p_{0}\right)\;\alpha^{2}\left\{\beta -(1-p_{0})\;\right\}$$

(7) $$\kappa_{3}=\left(1-p_{0}\right)\;\alpha^{3}\left\{\beta^{3}-3\left(1-p_{0}\right)\;\beta +2{\left(1-p_{0}\right)}^{2}\right\}$$

with:

(8) $$\alpha =\exp\left(\mu +\frac{1}{2}\sigma^{2}\right)$$

(9) $$\beta =\exp\left(\sigma^{2}\right)$$

Even if it is not necessarily the best possible definition, in this article, skewness is classically defined as:

(10) $$\gamma_{1}=E\left\{{\left(\frac{y-\kappa_{1}}{\kappa_{2}^{1/2}}\right)}^{3}\right\}=\frac{\kappa_{3}}{\kappa_{2}^{3/2}}$$

**Fig. 1:**
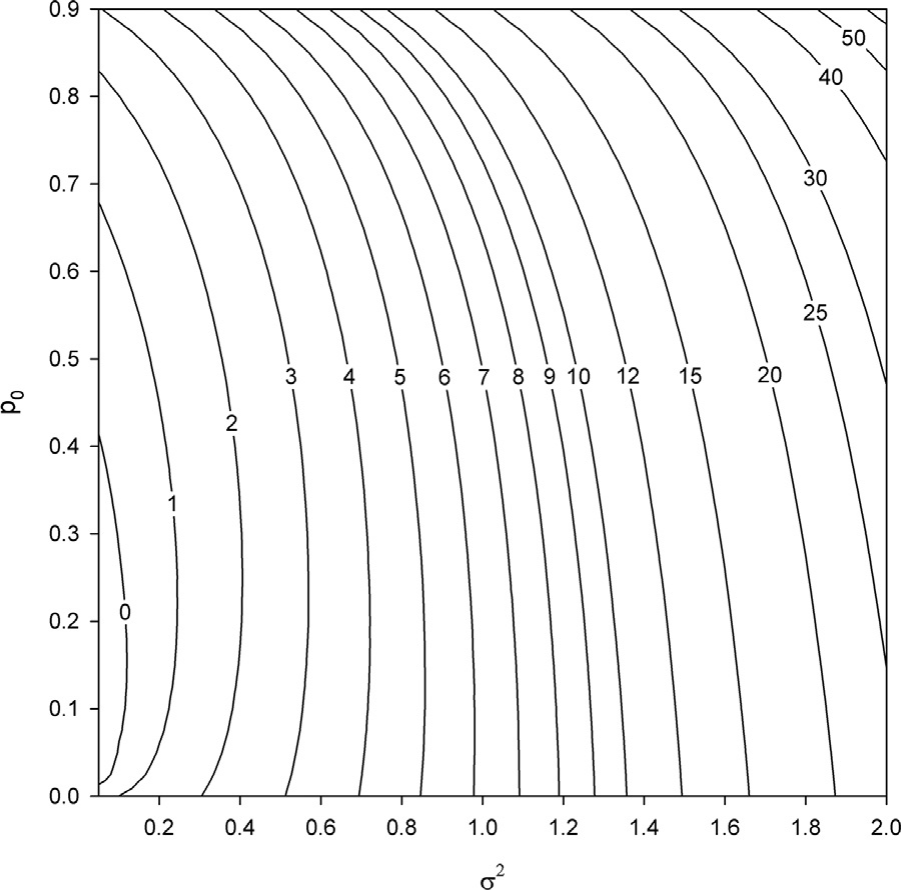
Skewness $\gamma_{1}$ for the delta-lognormal distribution as a function of $p_{0}$ and $\sigma^{2}$. For maximum bounds of the parameter space considered on the figure ($p_{0}=0.9,\sigma^{2}=2$), the skewness is $\gamma_{1}≃64.5$.

The skewness $\gamma_{1}$ of the delta-lognormal distribution increases dramatically as $\sigma^{2}$ increases. For small values of $\sigma^{2}$, as $p_{0}$ increases, $\gamma_{1}$ first decreases and then increases. For $\sigma^{2}>1$ approximately, as $p_{0}$ increases, $\gamma_{1}$ only increases (Fig. 1).

### Relative efficiency assessement

In the context of a finite population, one can indifferently consider the mean ${\bar{y}}_{U}$ or the total $t_{U}$ as the quantity of interest. For the finite population that has actually been sampled, these quantities have fixed values that one would be able to know exactly if s=U (ignoring possible measure or observation errors). Under a superpopulation model ξ, these statistics are random variables whose values one wants to predict. In an infinite population (superpopulation), one is interested in estimating the expectation $E\left(y\right)=\kappa_{1}$.

For delta-lognormal data, depending on the level of skweness of the distribution, the question arises of the gain in precision that can be achieved by relying on the *uniformly minimum-variance unbiased estimator* (UMVUE) compared to using the sample mean ${\bar{y}}_{s}$, either for estimating $\kappa_{1}$ or for predicting ${\bar{y}}_{U}$ (or equivalently, $t_{U}$). In other words, one may compare the situation where the shape of the distribution is known, to the situation where it is unknown (or known but not taken into account), first in the case of an infinite population (estimation context), then in that of a finite population (prediction context).

In the estimation context, Aitchison and Brown [1, p. 98, Fig. 9.1] provided relative efficiency results only for $p_{0}=0.5$ and for the degenerate case of the lognormal distribution ($p_{0}=0$), using a variance approximation (see the validation section). By doing so, the sample size n is disregarded in the relative efficiency assessment. Shimizu [2] did not document the relative efficiency in the case of the delta-lognormal distribution. Smith [4] considered the relative efficiency for $p_{0}=0.1$ and p=0.5, for very small sample sizes, using exact or approximate variance. To our knowledge, the relative efficiency assessment in the prediction context has not been documented yet.

In this technical article, after providing a compendium of fundamental formulas for UMVU estimation in the case of the delta-lognormal distribution, we document the relative efficiency more thoroughly than in the past by considering both the estimation and prediction contexts, taking into account the sample size (and the finite population size in the prediction context), and varying the probabilitiy of getting a zero value up to $p_{0}=0.9$. In all cases we use the exact expression of the variance of the estimator (or predictor).

## Estimation context

### Unknown shape of the distribution

Let s be a sample of size n drawn by random sampling from an infinite population of unknown shape. The unbiased estimator of $\kappa_{1}$ is the sample mean ${\bar{y}}_{s}$ and its sampling variance is written as:

(11) $$V\left({\bar{y}}_{s}\right)=\frac{\kappa_{2}}{n}$$

where $\kappa_{2}$ is estimated without bias by:

(12) $$S_{s}^{2}=\frac{1}{n-1}\sum_{i\in s}{\left(y_{i}-{\bar{y}}_{s}\right)}^{2}$$

The sampling variance is then estimated without bias by:

(13) $$\hat{V}\left({\bar{y}}_{s}\right)=\frac{S_{s}^{2}}{n}$$

### Known shape of the distribution

When y is distributed according to a delta-lognormal distribution, $\kappa_{1}$ can be estimated by the UMVUE [1, p. 97, Eq. (9.54)] (typo corrected); [4]:

(14) $${\hat{\kappa }}_{1}=\left\{ \begin{array}{cc} \left(1-{\hat{p}}_{0}\right)\exp\left({\bar{y}}_{\text{ln}}\right)\;g_{n_{1}-1}\;\left(\frac{1}{2}s_{\text{ln}}^{2}\right) & \;n_{1}>1\\ \frac{y_{s_{1}}}{n} & \;n_{1}=1\\ 0 & \;n_{1}=0 \end{array}\right.$$

with:

(15) $${\bar{y}}_{\text{ln}}=\frac{1}{n_{1}}\sum_{i\in s_{1}}\ln y_{i}$$

(16) $$s_{\text{ln}}^{2}=\frac{1}{n_{1}-1}\sum_{i\in s_{1}}{\left(\ln y_{i}-{\bar{y}}_{\text{ln}}\right)}^{2}$$

and $g_{m}\;\left(t\right)$ an infinite series introduced by Finney [5, Eq. (10)], which can be written as [6, Eq. (1.2)], [7, Eq. (3)]:

(17) $$g_{m}\;\left(t\right)=\sum_{j=0}^{\infty }\frac{1}{j!}\frac{m+2j}{m}{\left(\frac{m}{m+1}t\right)}^{j}\prod_{i=1}^{j}\frac{m}{m+2i}$$

The exact variance of ${\hat{\kappa }}_{1}$ (14) was provided by Smith [4, Eq. (6)]. The function $g_{m}\left(t\right)$ belongs to the class of generalized hypergeometric functions and can be written as a particular instance of the confluent hypergeometric limit function (here denoted as ${}_{0}F_{1}$) as [6, Eq. (2.1)]:

(18) $$g_{m}\;\left(t\right)={}_{0}F_{1}\left(\frac{m}{2};\frac{m^{2}\;t}{2(m+1)}\right)$$

with [8, Eq. (4)], [9, p. 333, Eq. (16.3.1)]:

(19) $${}_{0}F_{1}\left(a;z\right)=\sum_{j=0}^{\infty }\frac{z^{j}}{j!\;{\left(a\right)}_{j}}$$

where ${\left(a\right)}_{j}$ is the notation used in special function theory for the rising factorial:

(20) $${\left(a\right)}_{j}=\left\{ \begin{array}{cc} 1 & \;j=0\\ \prod_{i=0}^{j-1}\left(a+i\right) & \;j\geq 1 \end{array}\right.$$

Note that the numerical evaluation of special functions ${}_{0}F_{1}\left(a;z\right)$ and $g_{m}\;\left(t\right)$ is addressed later in the article.

Using the confluent hypergeometric limit function, according to Shimizu [2, p. 50], estimators ${\hat{\kappa }}_{1}$ and ${\hat{\kappa }}_{2}$ can be written, respectively, as:

(21) $${\hat{\kappa }}_{1}=\left\{ \begin{array}{cc} \left(1-{\hat{p}}_{0}\right)\exp\left({\bar{y}}_{\text{ln}}\right)\;{}_{0}F_{1}\;\left(\frac{n_{1}-1}{2};\frac{{\left(n_{1}-1\right)}^{2}}{4n_{1}}s_{\text{ln}}^{2}\right) & \;n_{1}>1\\ \frac{y_{s_{1}}}{n} & \;n_{1}=1\\ 0 & \;n_{1}=0 \end{array}\right.$$

and

(22) $${\hat{\kappa }}_{2}=\left\{ \begin{array}{cc} \left(1-{\hat{p}}_{0}\right)\exp\left(2{\bar{\text{y}}}_{\text{ln}}\right)[{}_{0}F_{1}\;\left(\frac{n_{1}-1}{2};\frac{{\left(n_{1}-1\right)}^{2}}{n_{1}}s_{\text{ln}}^{2}\right) & \\ \;\;\;\;\;-\left(\frac{n_{1}-1}{n-1}\right){}_{0}F_{1}\;\left(\frac{n_{1}-1}{2};\frac{\left(n_{1}-1\right)\left(n_{1}-2\right)}{2n_{1}}s_{\text{ln}}^{2}\right)] & n_{1}>1\\ \frac{y_{s_{1}}^{2}}{n} & n_{1}=1\\ 0 & n_{1}=0 \end{array}\right.$$

The exact variance of ${\hat{\kappa }}_{1}$ is [10, Remark 3.1], [2, pp. 50-51]:

(23) V(κ^1)=α2[1n2∑j=2n(nj)(1−p0)jp0n−jj2exp(σ2j)×0F1(j−12;(j−1)24j2σ4)−(1−p0)2]

Equivalent approximations of $V({\hat{\kappa }}_{1})$ were given by Shimizu [2, p. 51] and Aitchison and Brown [1, p. 99, Eq. (9.58)] (see the validation section); in this article we use the exact expression (23), which translates into Algorithm 1, using Algorithm 2 [11].

**Algorithm 1:**
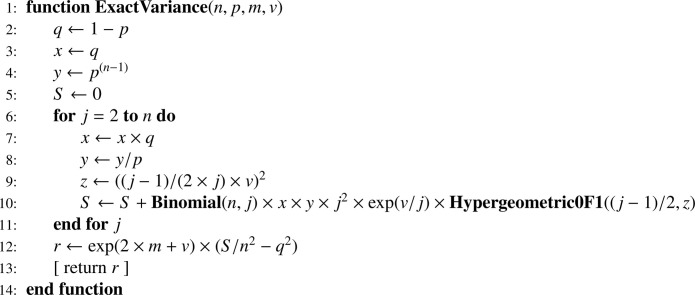
Exact variance $V({\hat{\kappa }}_{1})$(23) with n the sample size, p, m, v the values for parameters $p_{0}$, μ, $\sigma^{2}$, respectively.

**Algorithm 2:**
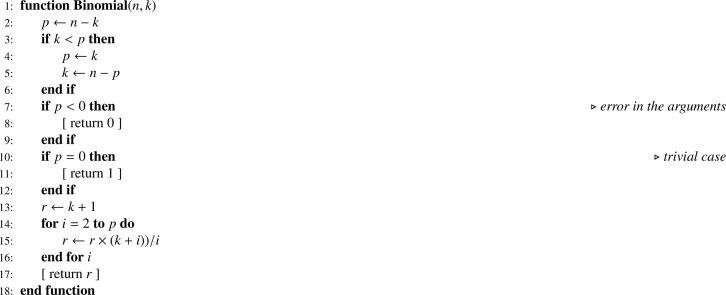
Iterative computation of the binomial coefficient.

Pennington [12] has provided the UMVUE for the variance $V({\hat{\kappa }}_{1})$ expressed with the function $g_{m}\;\left(t\right)$ [12, Eq. (4)]; for the sake of homogeneity, we present it here expressed using the confluent hypergeometric limit function:

(24) $$\hat{\text{V}}\left({\hat{\kappa }}_{1}\right)=\left\{ \begin{array}{cc} \begin{array}{cc} & \left(1-{\hat{p}}_{0}\right)\exp\left(2{\bar{\text{y}}}_{\text{ln}}\right)[\left(1-{\hat{p}}_{0}\right)\;{}_{0}F_{1}^{2}\;\left(\frac{n_{1}-1}{2};\frac{{\left(n_{1}-1\right)}^{2}}{4n_{1}}s_{\text{ln}}^{2}\right)\\ & -\left(\frac{n_{1}-1}{n-1}\right){}_{0}F_{1}\;\left(\frac{n_{1}-1}{2};\frac{\left(n_{1}-1\right)\left(n_{1}-2\right)}{2n_{1}}s_{\text{ln}}^{2}\right)] \end{array} & \;n_{1}>1\\ {\left(\frac{y_{s_{1}}}{n}\right)}^{2} & \;n_{1}=1\\ 0 & \;n_{1}=0 \end{array}\right.$$

#### Relative efficiency

We compare the precision of the estimator for $\kappa_{1}$ according to whether the distribution shape is unknown or known. It is expected that taking into account the knowledge of the distribution shape will lead to a gain in precision. The relative efficiency is defined in the same way as that used by Aitchison and Brown [1, p. 99, Eq. (9.62)]:

(25) $${\text{eff}}_{1}=\frac{V({\hat{\kappa }}_{1})}{V({\bar{y}}_{s})}$$

that is, taking into account the shape of the distribution leads to a gain in precision, which is higher when ${\text{eff}}_{1}$ is low ($V\left({\hat{\kappa }}_{1}\right)<V\left({\bar{y}}_{s}\right)$). Hence, we quantify the gain in precision by expressing it as a function of $p_{0}$, $\sigma^{2}$ (or, equivalently, σ) and n. The parameter μ vanishes through the elimination of the $\alpha^{2}$ term that appears in the numerator and denominator of the relative efficiency.

**Fig. 2:**
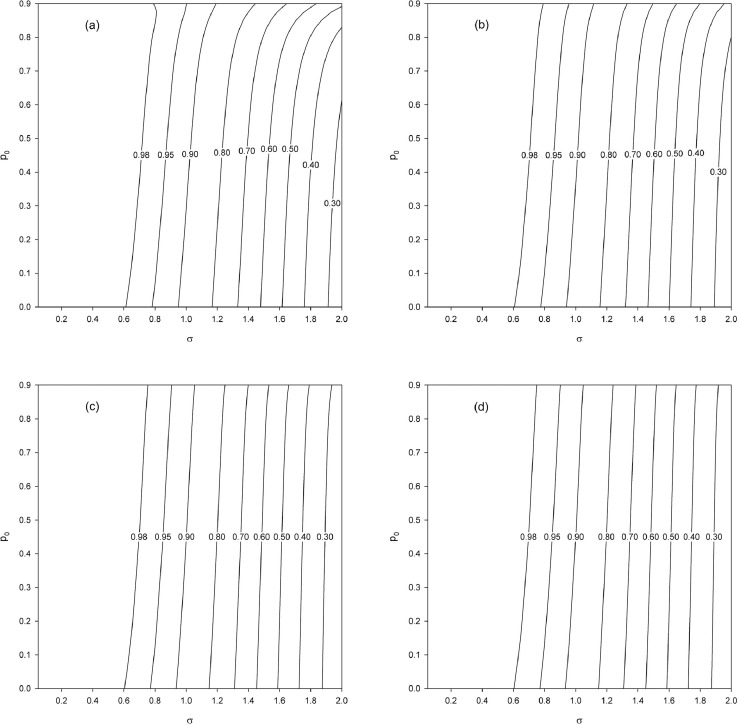
Relative efficiency ${\text{eff}}_{1}$ as a function of $p_{0}$ and σ for different sample sizes (n). (a) n=50. (b) n=100. (c) n=500. (d) n=1000.

We vary $p_{0}=0\left(0.025\right)0.9$, σ=0.05(0.05)2 and n=50,100,500,1000. The results show that the gain in precision increases substantially (${\text{eff}}_{1}$ decreases) as σ increases and increases more weakly — for a fixed $\left(p_{0},\sigma \right)$ point — as n increases (Fig. 2). For a fixed σ value, the gain decreases (${\text{eff}}_{1}$ increases) as $p_{0}$ increases, and this is all the more pronounced when n is small (see Fig. 2a). This phenomenon diminishes as n increases (see Fig. 2b–d). Finally, we note a small difference between n=500 (Fig. 2c) and n=1000 (Fig. 2d), which suggests a convergence of ${\text{eff}}_{1}$ toward its asymptotic limit.

**Fig. 3:**
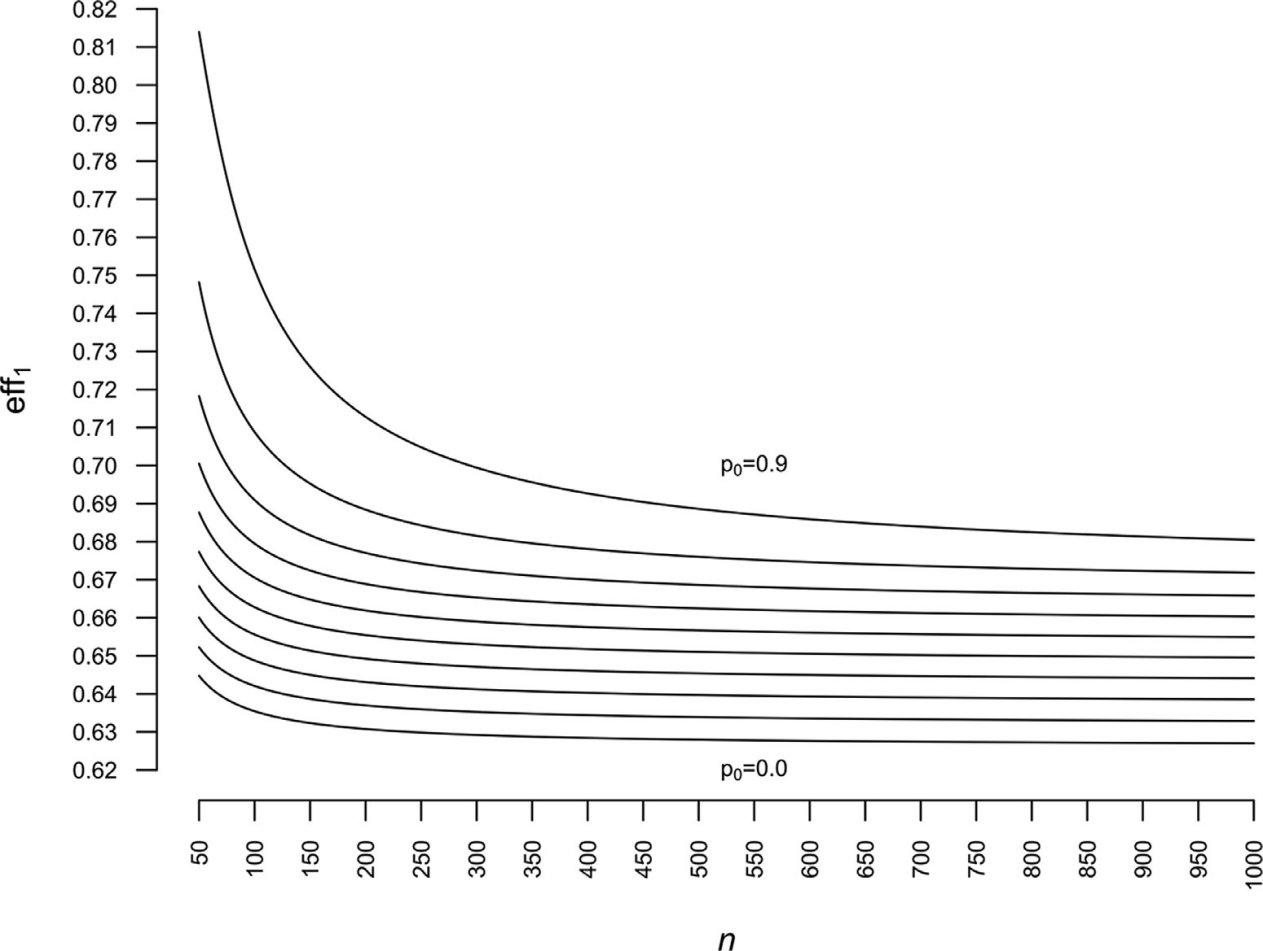
Relative efficiency ${\text{eff}}_{1}$ as a function of sample size (n) for $\sigma^{2}=2$ and $p_{0}=0.0\left(0.1\right)0.9$.

To illustrate the speed of convergence of ${\text{eff}}_{1}$ toward its asymptotic limit, we set $\sigma^{2}=2$, and we repeat the calculations for $p_{0}=0.0\left(0.1\right)0.90$ and 50≤n≤1000. The asymptotic limit and the speed of convergence of ${\text{eff}}_{1}$ depend on $p_{0}$; the lower $p_{0}$ is, the higher the efficiency gain and the speed of convergence (Fig. 3).

## Prediction context

By denoting r=U−s, the total defined on the population can be written as:

(26) $$t_{U}=\sum_{i\in U}y_{i}=\underset{t_{s}}{\underset{︸}{\sum_{i\in s}y_{i}}}+\underset{t_{r}}{\underset{︸}{\sum_{i\in r}y_{i}}}$$

that is the sum of the totals defined over the sample ($t_{s}$) and the remaining part in the population ($t_{r}$). A predictor of $t_{U}$ can be written as:

(27) $${\tilde{t}}_{U}=\sum_{i\in s}y_{i}+\underset{{\tilde{t}}_{r}}{\underset{︸}{\sum_{i\in r}{\tilde{y}}_{i}}}$$

Consider a simple mean model where the random variables $y_{i}$ (i=1,⋯,N) have the same expectations and variances and are not correlated, that is:

(28a) $$E\left(y_{i}\right)=\kappa_{1}$$

(28b) $$V\left(y_{i}\right)=\kappa_{2}$$

(28c) $$\mathrm{Cov}\left(y_{i},y_{j}\right)=0\;\;\left(i\neq j\right)$$

The empirical predictor is written as:

(29) $${\tilde{t}}_{U}=\sum_{i\in s}y_{i}+\underset{{\tilde{t}}_{r}}{\underset{︸}{\left(N-n\right)\hat{E}\left(y_{i}\right)}}$$

Predictor (29) is model-unbiased; that is, if the model is correct, then $E({\tilde{t}}_{U}-t_{U})=0$. The variance of the prediction error is obtained as:

(30) $$V\left({\tilde{t}}_{U}-t_{U}\right)=V\left({\tilde{t}}_{r}-t_{r}\right)=V\left({\tilde{t}}_{r}\right)+V\left(t_{r}\right)-2\underset{0}{\underset{︸}{\mathrm{Cov}({\tilde{t}}_{r},t_{r})}}$$

The ξ-covariance between ${\tilde{t}}_{r}$ and $t_{r}$ is zero since: (i) ${\tilde{t}}_{r}$ is a function of the set of values in s ($\left\{y_{i},i\in s\right\}$), not of the set of values in r ($\left\{y_{i},i\in r\right\}$) which we do not know; and (ii) the two sets of values $\left\{y_{i},i\in s\right\}$ and $\left\{y_{i},i\in r\right\}$ are uncorrelated under the model (see (28c)).

### Distribution of unknown shape

Predictor (29) can be written as:

(31) $${\tilde{t}}_{U}^{\;\text{exp}}=\sum_{i\in s}y_{i}+\left(N-n\right){\bar{y}}_{s}=n{\bar{y}}_{s}+\left(N-n\right){\bar{y}}_{s}=N{\bar{y}}_{s}$$

which we can designate as an *expansion predictor* [13]. From relation (30), the prediction error variance of $\left({\tilde{t}}_{U}^{\;\text{exp}}-t_{U}\right)$ is obtained as:

(32) $$V({\tilde{t}}_{U}^{\;\text{exp}}-t_{U})={\left(N-n\right)}^{2}\left[V\left({\bar{y}}_{s}\right)+V\left({\bar{y}}_{r}\right)\right]$$

(33) $$\;={\left(N-n\right)}^{2}\left(\frac{1}{n}+\frac{1}{N-n}\right)\kappa_{2}$$

(34) $$\;=N^{2}\left(1-\frac{n}{N}\right)\frac{\kappa_{2}}{n}$$

The predictor of the mean is ${\bar{y}}_{s}$, and its prediction error variance is $V\left({\bar{y}}_{s}-{\bar{y}}_{U}\right)=\left(1-n/N\right)\kappa_{2}/n$. It follows the limit:

(35) $$\lim_{N\to \infty }V\left({\bar{y}}_{s}-{\bar{y}}_{U}\right)=V\left({\bar{y}}_{s}\right)$$

For prediction error variance (34), an unbiased estimator is obtained by substituting estimator ${\hat{\kappa }}_{2}$ (22) for parameter $\kappa_{2}$.

### Distribution of known shape

Predictor (29) can be written as:

(36) $${\tilde{t}}_{U}^{\;\text{mvu}}=\sum_{i\in s}y_{i}+\left(N-n\right){\hat{\kappa }}_{1}$$

From relation (30), the prediction error variance (${\tilde{t}}_{U}^{\;\text{mvu}}-t_{U})$ is obtained as:

(37) $$V({\tilde{t}}_{U}^{\;\text{mvu}}-t_{U})={\left(N-n\right)}^{2}\left[V\left({\hat{\kappa }}_{1}\right)+V\left({\bar{y}}_{r}\right)\right]$$

(38) $$\;=\left(N-n\right)\left[\left(N-n\right)V\left({\hat{\kappa }}_{1}\right)+\kappa_{2}\right]$$

where the variance $V({\hat{\kappa }}_{1})$ is given by expression (23). The mean predictor is ${\tilde{\bar{y}}}_{U}=N^{-1}{\tilde{t}}_{U}^{\;\text{mvu}}$ (e.g., [14, Eq. (3)]), and its prediction error variance is $V\left({\tilde{\bar{y}}}_{U}-{\bar{y}}_{U}\right)=N^{-2}V\left({\tilde{t}}_{U}^{\;\text{mvu}}-t_{U}\right)$. It follows the limits:

(39) $$\lim_{N\to \infty }{\tilde{\bar{y}}}_{U}=\lim_{N\to \infty }\left[\frac{1}{N}\sum_{i\in s}y_{i}+\frac{\left(N-n\right)}{N}{\hat{\kappa }}_{1}\right]={\hat{\kappa }}_{1}$$

(40) $$\lim_{N\to \infty }V\left({\tilde{\bar{y}}}_{U}-{\bar{y}}_{U}\right)=\lim_{N\to \infty }\left[\frac{{\left(N-n\right)}^{2}}{N^{2}}V\left({\hat{\kappa }}_{1}\right)+\frac{\left(N-n\right)}{N^{2}}\kappa_{2}\right]=V\left({\hat{\kappa }}_{1}\right)$$

For prediction error variance (38), an unbiased estimator is obtained by substituting estimators $\hat{V}\left({\hat{\kappa }}_{1}\right)$ (24) and ${\hat{\kappa }}_{2}$ (22) for $V\left({\hat{\kappa }}_{1}\right)$ and $\kappa_{2}$, respectively.

#### Relative efficiency

We compare the precision of the $t_{U}$ prediction depending on whether the shape of the distribution is unknown or known. In addition to $p_{0}$, $\sigma^{2}$ and n, we must also vary the finite population size N. The relative efficiency is defined as:

(41) $${\text{eff}}_{2}=\frac{V({\tilde{t}}_{U}^{\;\text{mvu}}-t_{U})}{V({\tilde{t}}_{U}^{\;\text{exp}}-t_{U})}=\frac{V({\tilde{\bar{y}}}_{U}-{\bar{y}}_{U})}{V({\bar{y}}_{s}-{\bar{y}}_{U})}$$

From (35) and (40), we obtain:

(42) $$\lim_{N\to \infty }{\text{eff}}_{2}={\text{eff}}_{1}$$

**Fig. 4:**
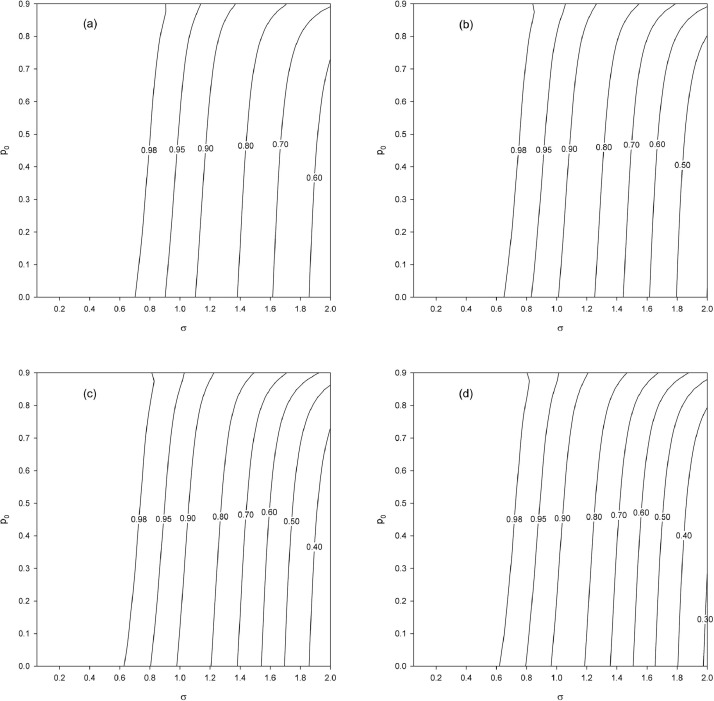
Relative efficiency ${\text{eff}}_{2}$ as a function of $p_{0}$ and σ for a fixed sample size (n=50) and different finite population sizes (N) and hence different sampling fractions (f). (a) N=125, f=0.4. (b) N=250, f=0.2. (c) N=500, f=0.1. (d) N=1000, f=0.05.

First, we examine the effect of the population size N for a fixed sample size n, which is equivalent to examining the effect of the sampling fraction f=n/N. We set n=50 and vary the sampling fraction as f=0.4,0.2,0.1,0.05 (N=125,250,500,1000). As before, we vary $p_{0}=0\left(0.025\right)0.9$ and σ=0.05(0.05)2. The obtained results (Fig. 4) show a smaller gain in precision than in the case of an infinite population (Fig. 2a). This finite population effect is less pronounced as f tends toward 0 (N→∞) since in that scenario the situation tends toward the asymptotic result of the case considered here, which corresponds to Fig. 2a.

**Fig. 5:**
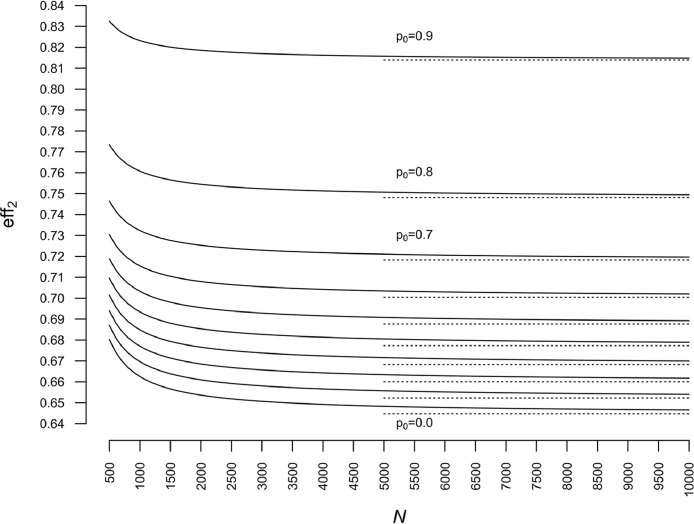
Relative efficiency ${\text{eff}}_{2}$ as a function of population size (N) for a fixed sample size (n=50), for $\sigma^{2}=2$ and $p_{0}=0.0\left(0.1\right)0.9$. Details in the text.

To illustrate the speed of convergence of ${\text{eff}}_{2}$ toward its asymptotic limit ${\text{eff}}_{1}$, we set $\sigma^{2}=2$, and we repeat the calculations for $p_{0}=0.0\left(0.1\right)0.90$, n=50 and 500≤N≤10000. As in the infinite population case, the asymptotic limit and the speed of convergence of ${\text{eff}}_{2}$ depend on $p_{0}$. The lower $p_{0}$ is, the higher the efficiency gain (${\text{eff}}_{2}$ decreases). In contrast, the speed of convergence becomes less important when $p_{0}$ decreases (Fig. 5). In practice, we can consider that we are almost at convergence as soon as N=5000 (or N=10000 if one is more conservative). Note that limit values correspond to the values for n=50 in Fig. 3 and are represented by dotted half lines in Fig. 5.

**Fig. 6:**
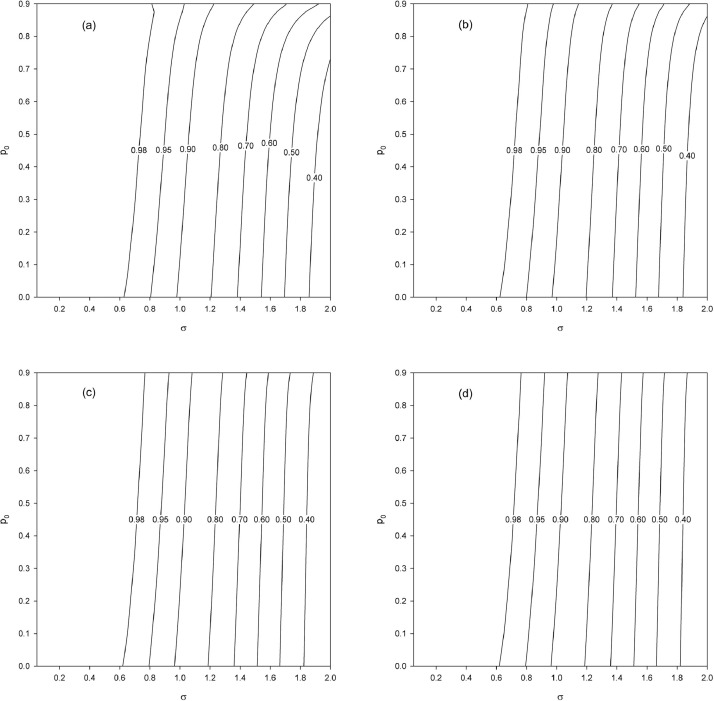
Relative efficiency ${\text{eff}}_{2}$ as a function of $p_{0}$ and σ for a fixed sampling fraction f=0.1 and increasing finite population (N) and sample (n) sizes. (a) N=500 (n=50). (b) N=1000 (n=100). (c) N=5000 (n=500). (d) N=10000 (n=1000).

Finally, we examine the gain in precision when n and N increase jointly, keeping the sampling fraction constant. For f=0.1, we increase the population size as N=500,1000,5000,10000 (n=50,100,500,1000). The obtained results (Fig. 6) show a gain in precision that increases (${\text{eff}}_{2}$ decreases) as N and n jointly increase. Note that Fig. 6a is the same as Fig. 4c (N=500, n=50). There is only a small difference between the case N=5000,n=500 (Fig. 6c) and the case N=10000,n=1000 (Fig. 6d), which suggests the convergence of ${\text{eff}}_{2}$ toward its asymptotic limit (in the sense that n and N jointly increase and for f=0.1).

## Numerical evaluation of the generalized hypergeometric functions

In this article, we need to numerically evaluate ${}_{0}F_{1}\left(a;z\right)$ ($a\in R_{+}^{*}$ and $z\in R_{+}^{*}$) — or equivalently $g_{m}\left(t\right)$ — to calculate $V({\hat{\kappa }}_{1})$ (23), which is directly involved in the relative efficiency ${\text{eff}}_{1}$ (25) and through formula (38) in the relative efficiency ${\text{eff}}_{2}$ (41).

### Evaluating ${}_{0}F_{1}\left(a;z\right)$ by recurrence

The confluent hypergeometric limit function (19) can be written as:

(43) $${}_{0}F_{1}\left(a;z\right)=1+\frac{z}{a}+\sum_{j=2}^{\infty }\frac{1}{\prod_{i=0}^{j-1}\left(a+i\right)}\times \frac{z^{j}}{j!}$$

which gives the recurrence relation:

(44) $$\begin{array}{ccc} A_{1}=\frac{z}{a} & \;\; & S_{1}=1+A_{1}\\ A_{j}=A_{j-1}\times \frac{1}{a+j-1}\times \frac{z}{j} & \;\; & S_{j}=S_{j-1}+A_{j}\;\;\text{for}\;j=2,3,\ldots ,k \end{array}$$

and translates into Algorithm 3.

**Algorithm 3:**
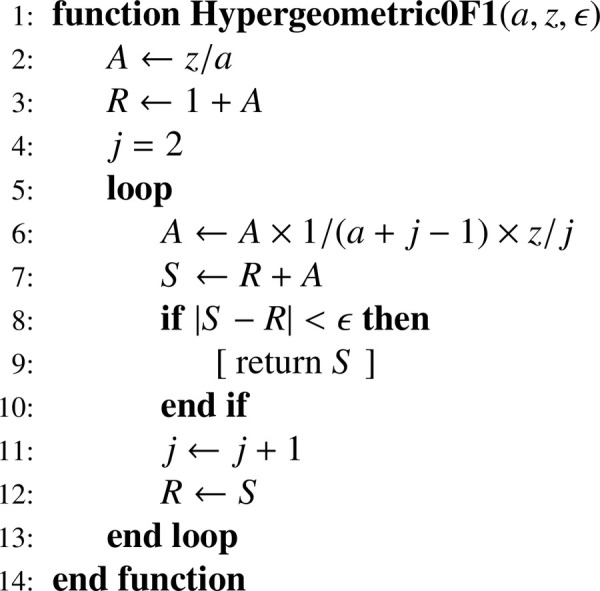
Confluent hypergeometric limit function ${}_{0}F_{1}$.

The series is infinite, but in practice, it is only required to compute the sum until it reaches a level of convergence considered sufficient; for example, with the convergence criterion $|S_{j}-S_{j-1}|<\epsilon$, we used $\epsilon =10^{-10}$. Another way to proceed is to determine the number of terms necessary to reach the precision allowed by the computer at hand (see [15, pp. 88-89]). Computing successive terms of the series by means of the recurrence relation is a computational method often used by default (e.g., [16, p. 99]). For other methods, the interested reader is referred to [17], [18].

### Evaluating $g_{m}\;\left(t\right)$ by recurrence

One can proceed in the same way as previously for the function $g_{m}\;\left(t\right)$ (17), which can be written as (see, for example, [4, Eq. (1)], with $m=n_{1}-1$):

(45) $$g_{m}\;\left(t\right)=1+\frac{m}{m+1}\times t+\sum_{j=2}^{\infty }\frac{m^{2j-1}}{{\left(m+1\right)}^{j}\;\prod_{i=2}^{j}\left(m+2\left(i-1\right)\right)}\times \frac{t^{j}}{j!}$$

which gives the recurrence relation:

(46) $$\begin{array}{ccc} A_{1}=\frac{m}{m+1}\times t & \;\; & S_{1}=1+A_{1}\\ A_{j}=A_{j-1}\times \frac{m^{2}}{\left(m+1)(m+2(j-1)\right)}\times \frac{t}{j} & \;\; & S_{j}=S_{j-1}+A_{j}\;\;\text{for}\;j=2,3,\ldots ,k \end{array}$$

and translates into Algorithm 4.

**Algorithm 4:**
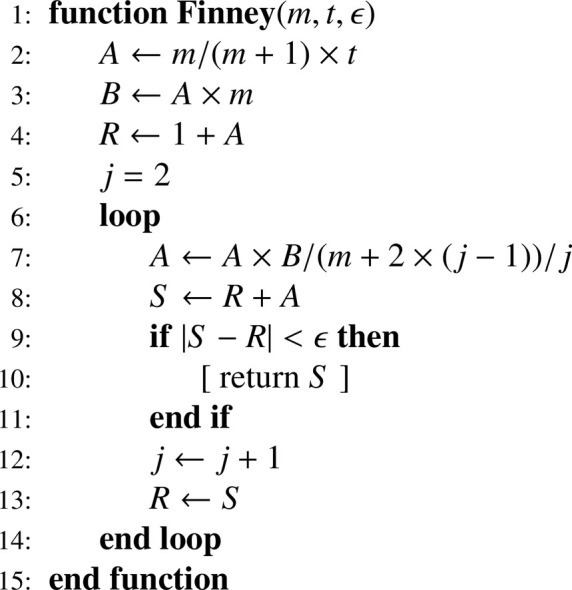
Finney’s generalized hypergeometric function $g_{m}\left(t\right)$

## Validation

In this section: (i) we check the calculation accuracy of the confluent hypergeometric limit function ${}_{0}F_{1}\left(a;z\right)$ required for computing $V({\hat{\kappa }}_{1})$ using Algorithm 1; (ii) we assess the correctness of Algorithm 1 by comparing its results against those of Monte Carlo simulations; (iii) we justify the use of the exact expression for the variance $V({\hat{\kappa }}_{1})$ (23) for accurately documenting the relative efficiency when considering ${\hat{\kappa }}_{1}$ in the estimation or prediction contexts.

### Precision of ${}_{0}F_{1}\left(a;z\right)$ evaluated by recurrence

We verify that the calculations performed in this article — using double-precision floating-point arithmetic — by implementing the computation of ${}_{0}F_{1}\left(a;z\right)$ through Algorithm 3 with $\epsilon =10^{-10}$ have sufficient numerical accuracy. To do this, we first examine the ranges of variation of parameter a and argument z and then compare the results of our procedure with reference values.

The examination of $V({\hat{\kappa }}_{1})$ (23) shows that the minimal value that a can take in this expression is a=0.5 and that for the maximal value n=1000 used in this article, we have a≃500. Besides, we may consider 0<z≤1000 for covering the situations addressed in [19] and similar future examples.

For a=0.5, it is possible to use a particular representation of ${}_{0}F_{1}\left(a;z\right)$ [20, p. 594, Table 7.13.1 (row 5, column 2)]:

(47) $${}_{0}F_{1}\left(0.5;z\right)=\cosh\left(2\sqrt{z}\right)$$

with cosh=(exp(z)+exp(−z))/2. For z=0.01(0.01)1, we obtain an absolute error less than $10^{-12}$ (at most k=8 iterations are required). For z=1(1)1000, we obtain a relative error less than $10^{-12}$. To gain an idea of the order of magnitude, ${}_{0}F_{1}\left(0.5;1000\right)≃1.466103955241\times 10^{27}$ (at most k=69 iterations are required).

For a>0.5, we use as reference values those returned by the function Hypergeometric0F1[a,z] of Mathematica^Ⓡ^. For a=5(5)500 and z=0.01(0.01)1, we obtain an absolute error less than $10^{-12}$ (at most k=7 iterations are required). For a=5(5)500 and z=10(10)1000, we obtain a relative error less than $10^{-11}$ (at most k=67 iterations are required).

### Monte Carlo simulations

We simulate K random samples of size n from a delta-lognormal distribution of parameters $p_{0}$, μ=1.5 and σ=2, and we compute the UMVUE (21) for each sample. This results in K values ${\hat{\kappa }}_{1}$ from which we calculate Monte Carlo approximations of expectation (denoted as $E_{\text{MC}}\left({\hat{\kappa }}_{1}\right)$) and variance (denoted as $V_{\text{MC}}\left({\hat{\kappa }}_{1}\right)$). Then, we can compare these Monte Carlo approximations to their theoretical values, that is, $\kappa_{1}$ — since ${\hat{\kappa }}_{1}$ is unbiased — and $V({\hat{\kappa }}_{1})$, respectively. We vary n=50,100,500,1000 and $p_{0}=0.1,0.5,0.9$. For accurate Monte Carlo approximations, we set $K=10^{8}$ for n=50,100 and $K=10^{7}$ for n=500,1000. Table 1 shows a very good agreement between the Monte Carlo approximations and the calculated theoretical values. We conclude that, for 50≤n≤1000 and $p_{0}\leq 0.9$, one can trust Algorithm 1.

**Table 1:** Results of the Monte Carlo simulations to assess the correctness of Algorithm 1. Details in the text.

$p_{0}$	n	K	$\kappa_{1}$	$E_{\text{MC}}\left({\hat{\kappa }}_{1}\right)$	$V({\hat{\kappa }}_{1})$	$V_{\text{MC}}\left({\hat{\kappa }}_{1}\right)$
0.1	50	$10^{8}$	29.8039	29.8039	268.1132	268.2039
0.1	100	$10^{8}$	29.8039	29.8027	126.5944	126.5823
0.1	500	$10^{7}$	29.8039	29.8032	24.1667	24.1690
0.1	1000	$10^{7}$	29.8039	29.8028	12.0127	12.0134
0.5	50	$10^{8}$	16.5577	16.5584	167.2448	167.2557
0.5	100	$10^{8}$	16.5577	16.5568	75.8297	75.8418
0.5	500	$10^{7}$	16.5577	16.5574	13.9909	13.9922
0.5	1000	$10^{7}$	16.5577	16.5565	6.9244	6.9199
0.9	50	$10^{8}$	3.3115	3.3113	61.8555	61.3674
0.9	100	$10^{8}$	3.3115	3.3115	22.3205	22.3108
0.9	500	$10^{7}$	3.3115	3.3108	3.1210	3.1220
0.9	1000	$10^{7}$	3.3115	3.3112	1.4862	1.4867

### Variance approximation of $V({\hat{\kappa }}_{1})$

An approximation of $V({\hat{\kappa }}_{1})$ in $O(n^{-2})$ is [2, p. 51]:

(48) $$V_{\text{app}}\left({\hat{\kappa }}_{1}\right)=\frac{1}{n}\left(1-p_{0}\right)\left[p_{0}+\frac{1}{2}\sigma^{2}\left(\sigma^{2}+2\right)\right]\exp\left(2\mu +\sigma^{2}\right)$$

**Fig. 7:**
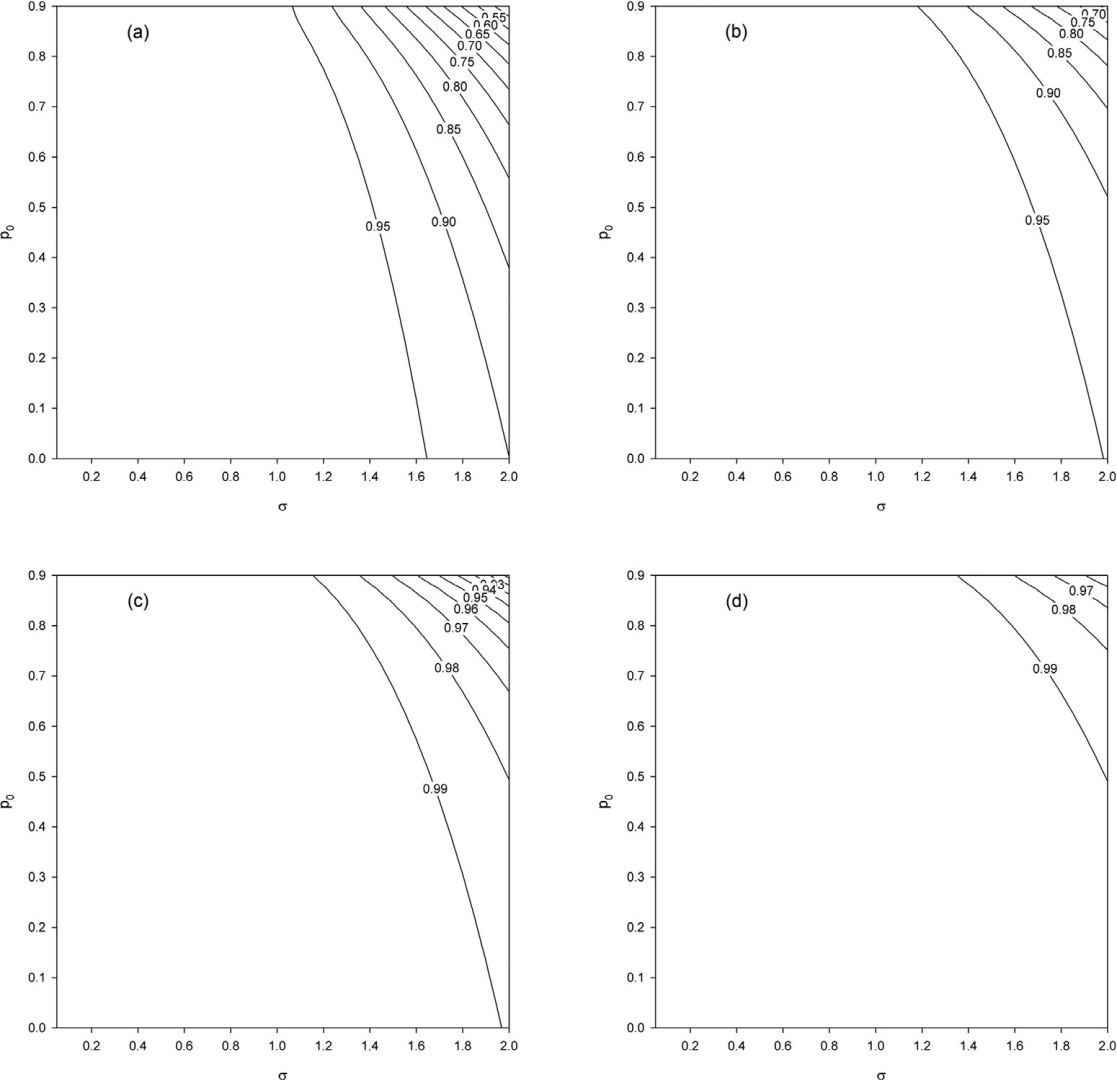
Ratio $V_{\text{app}}\left({\hat{\kappa }}_{1}\right)/V\left({\hat{\kappa }}_{1}\right)$ as a function of $p_{0}$ and σ for different sample sizes (n). (a) n=50. (b) n=100. (c) n=500. (d) n=1000.

Approximation (48) is algebraically equivalent to the approximation given by Aitchison and Brown [1, p. 99, Eq. (9.58)]. Aitchison & Brown indicated that this approximation is given for large n and $p_{0}$ substantially less than 1, without further details. The quality of approximation (48) depends on the sample size n and parameters $p_{0}$ and $\sigma^{2}$. To specify the domains of use of the approximation, we compute the ratio $V_{\text{app}}\left({\hat{\kappa }}_{1}\right)/V\left({\hat{\kappa }}_{1}\right)$ for p=0(0.025)0.9, σ=0.05(0.05)2.0 and n=50,100,500,1000 (Fig. 7). The approximation improves when σ and $p_{0}$ are small, and for the same fixed $\left(p_{0},\sigma \right)$ point, the accuracy increases as the sample size n increases. Thus, accurate calculations for great $p_{0}$ (i.e., here up to $p_{0}=0.9$) and $\sigma^{2}$ values requires using formula (23) (translated into Algorithm 1) instead of approximation (48), unless n is sufficiently large.

## Additional information

Pennington [12] introduced the use of the delta-lognormal distribution in marine biology to estimate mean abundance more efficiently than can be achieve with the sample mean in the case of highly skewed distributions. The article by Pennington [12] is widely cited in the literature (603 citations according to Google Scholar, at the time of writing this article). Regarding the robustness of this approach, the reader is referred to Myers and Pepin [21,^22^], Syrjala [23] and Christman [24]. In the context of the mean (or total) prediction, see the recent contribution [19].

## Declaration of Competing Interest

The author declare that he has no known competing for financial interests or personal relationships that could have appeared to influence the work reported in this paper.
